# Investigating the Catalytic Effects of Ni and Fe Doping on CaO for Enhancing the Glycerol Valorization into Lactic Acid and 1,3-Propanediol

**DOI:** 10.1021/acsomega.5c13429

**Published:** 2026-05-02

**Authors:** Mohd Mohsin Ikram, Jitendra Carpenter, Virendra Kumar Saharan

**Affiliations:** 1 Department of Chemical Engineering, 193160 Malaviya National Institute of Technology, Jaipur, Rajasthan 302017, India; 2 125853 Manipal Institute of Technology, Manipal Academy of Higher Education, Manipal, Karnataka 576104, India

## Abstract

This study presents the synthesis of a waste-derived catalyst for the efficient glycerol valorization into value-added products, contributing to waste valorization, resource recovery, circular economy, and sustainable chemical production. The valorization of glycerol into lactic acid has been investigated using Fe- and Ni-doped calcium oxide (CaO) catalyst synthesized from marble waste through thermal decomposition. The catalysts were calcined at 500 °C and thoroughly characterized using Brunauer–Emmett–Teller (BET) surface area analysis, X-ray diffraction (XRD), scanning electron microscopy with energy-dispersive X-ray spectroscopy (SEM-EDS), transmission electron microscopy (TEM), and Fourier-transform infrared spectroscopy (FTIR). The optimized catalyst, designated as NiO·Fe_2_O_3_/CaO, achieved a lactic acid yield of 20.7 mol % under reaction conditions of 10 g of catalyst, a temperature of 260 °C, and a reaction time of 150 min. A glycerol conversion of 68.5% was recorded. Secondary products formed during the reaction included 1,3-propanediol, formic acid, and acetic acid, with their maximum yields being 8.45 mol %, 2.20 mol %, and 1.79 mol %, respectively, observed at 260 °C after 180 min. The lactic acid yield was found to be dependent on both temperature and reaction time, reaching a maximum at 260 °C and 150 min, after which it declined due to thermal degradation into lighter gases and oxygenated byproducts. These results highlight the potential of utilizing marble waste-derived catalysts for the sustainable and value-added transformation of glycerol.

## Graphical abstract

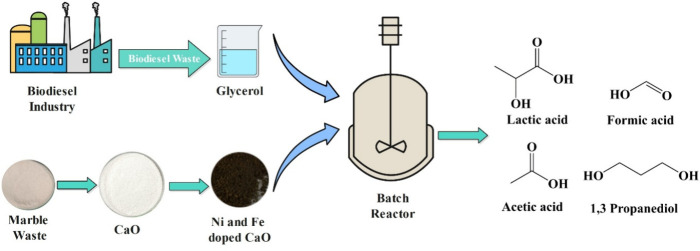

## 1 Introduction

As fossil fuel reserves continue to decline, the production of alternative energy sources such as biodiesel, bioethanol, and biogas has garnered significant attention. Biodiesel production, in particular, involves the transesterification of vegetable oils and animal fats, yielding fatty acid esters, commonly referred to as biodiesel, along with glycerol as a major coproduct^1^ The chemical process primarily involves the production of glycerol via the transesterification of triglycerides with an alcohol in the presence of a catalyst. This reaction produces fatty acid methyl esters (biodiesel) as the main product and glycerol as a significant byproduct. In the transesterification of triglycerides, the mass ratio of glycerol to biodiesel is approximately 1:10, indicating that for every 100 kg of biodiesel synthesized, about 10 kg of glycerol is generated.^2,3^ It is crucial to utilize this byproduct effectively; otherwise, its accumulation could pose significant storage challenges. With glycerol being produced in increasing quantities as a byproduct of biodiesel production, its valorization into value-added chemicals is essential to enhance both its utility and economic value. Chemically known as propane-1,2,3-triol, glycerol is the simplest naturally occurring trihydric alcohol, classified as a polyol due to its three hydroxyl groups, two primary and one secondary, which confer high reactivity and versatility.^4^ The efficiency and selectivity of glycerol conversion are strongly influenced by the acidity and spatial distribution of active sites on the catalyst.^5^ Numerous useful compounds, such as lactic acid, propanediol, syngas, hydrogen, acetals, alcohols, and polyglycerols, can be produced from glycerol. These conversion methods aid in the creation of sustainable and profitable products in addition to offering an efficient way to use glycerol.^6^

The distribution of desired products in glycerol conversion largely depends on several factors, including the type of catalyst, reactor configuration, operating temperature and pressure, catalyst-to-glycerol molar ratio, and the nature of the feedstock (pure glycerol or glycerol combined with other precursors). According to literature, glycerol can serve as a platform molecule for the synthesis of over 1,000 different chemicals.^7^ Achieving the desired product requires selecting an optimal catalyst and appropriate operating conditions. Among these products, lactic acid is a key compound with wide-ranging applications. It can be produced either by biological fermentation or catalytic conversion. Fermentation typically uses carbohydrates as feedstock but has drawbacks such as salt formation and complex downstream separation.^8^ This highlights the need to focus on catalytic methods which are less time-consuming and make use of glycerol a waste byproduct from the biodiesel industry.

For the glycerol valorization noble and non-noble catalyst can be used. For example, An Au–Pt doped CeO_2_ catalyst was employed in a batch reactor with NaOH at 373 K, achieved an 80% yield of lactic acid with 99% glycerol conversion, along with minor formation of glyceric acid, glycolic acid, oxalic acid, formic acid, and acetic acid.^9^ In another study, Au deposited on commercially available ultrastable zeolite-Y was employed under base-free conditions for the conversion of glycerol to methyl lactate, achieved 73% yield and 95% glycerol conversion after 10 h of reaction at temperature of 160 °C.^8^ The byproducts formed during glycerol conversion include glycerol methyl ethers such as 1-methyl ether, 1,3-dimethyl ether, and trimethyl ether. Key intermediates identified are glyceraldehyde (GLYA) and dihydroxyacetone (DHA).^1^ Overoxidation products include methyl glyoxal (MGLY) and dimethyl tartronate. Other byproducts observed are 5-hydroxy-1,3-dioxane, 4-hydroxymethyl-1,3-dioxolane, and glycerol formal.^10^ These findings indicate that when employing noble metal-based catalysts, the formation of multiple byproducts is often inevitable, posing challenges to selectivity and cost-effectiveness. Alternatively, non-noble transition metals doped onto basic supports like CaO have shown promising potential for selective lactic acid production from glycerol. For example, a NiO-doped CaO catalyst achieved a maximum lactic acid yield of 41.4 mol % at 89.3 mol % glycerol conversion after 1.5 h at 290 °C. The lactic acid yield was significantly influenced by the NiO loading, with 0.43 wt % NiO/CaO demonstrating the highest performance.^11^

Building on this approach, the present study focuses on the sustainable conversion of glycerol to lactic acid and 1,3-propanediol (1,3-PDO) using a Ni- and Fe-doped CaO (NiO.Fe_2_O_3_/CaO) catalyst synthesized from marble waste powder. The catalyst, characterized by enhanced basicity and redox properties, was applied in a batch reactor system. Key reaction parameters such as catalyst loading, temperature, and reaction time, were systematically optimized to maximize product yield and selectivity, demonstrating a cost-effective and environmentally conscious pathway for glycerol valorization.

## 2 Experimental Section

### 2.1 Materials

The calcium oxide was derived from marble waste powder which was collected from the marble waste dumping yard in Kishangarh, Rajasthan, India (26.6000°N, 74.9880°E). Glycerol (≥99.5%), absolute ethanol (≥99%) and Sulfuric acid (98%) of analytical grades were purchased from Merck. Ferric nitrate nonahydrate [Fe (NO_3_)_3_·9H_2_O] (≥98%, Loba Chemie Pvt. Ltd.) and nickel­(II) nitrate hexahydrate [Ni (NO_3_)_2_·6H_2_O] (≥98%, Chemical Drug House) were used as catalyst precursors. High-purity nitrogen gas (≥99.99%) was procured from Ankur Gas Agency, Jaipur, India. All chemicals were used without further purification. Deionized water was used for all experimental and analytical works.

### 2.2 Catalyst Preparation

Initially, marble waste powder (MWP) was sieved using a 60-mesh sieve to eliminate coarse particles and grit. The sieved powder was then thoroughly washed 4–5 times with demineralized water to remove any residual impurities. After washing, the MWP was dried and subsequently subjected to calcination in a muffle furnace at 850 °C for 2 h. Calcination is a crucial step that facilitates the thermal decomposition of MWP, resulting in the formation of oxides such as CaO, MgO, and SiO_2_. Equation 1–4) represent formation of CaO from marble waste powder.^12^ Acid treatment was carried out to remove insoluble silica impurities from the catalyst precursor. Following this treatment, the material was dried and subjected to calcination, resulting in the formation of purified CaO.

$${\mathrm{CaMgSi}}_{2}O_{6}\overset{\Delta \mathrm{at}850^{o}C}{\to }\mathrm{CaO}+\mathrm{MgO}+2{\mathrm{SiO}}_{2}$$ 1

$$\mathrm{CaMg}({\mathrm{CO}}_{3}{)}_{2}\overset{\Delta \mathrm{at}850^{o}C}{\to }\mathrm{CaO}+\mathrm{MgO}+2{\mathrm{CO}}_{2}↑$$ 2

$$\mathrm{CaO}+\mathrm{MgO}+2{\mathrm{SiO}}_{2}+4{\mathrm{HNO}}_{3\cdot }H_{2}O\to \mathrm{Ca}({\mathrm{NO}}_{3}{)}_{2}+\mathrm{Mg}({\mathrm{NO}}_{3}{)}_{2}+6H_{2}O+2{\mathrm{SiO}}_{2}$$ 3

$$\mathrm{Ca}({\mathrm{NO}}_{3}{)}_{2}+\mathrm{Mg}({\mathrm{NO}}_{3}{)}_{2}\overset{\Delta \mathrm{at}850^{o}C}{\to }\mathrm{CaO}+\mathrm{MgO}+4{\mathrm{NO}}_{2}+O_{2}$$ 4

NiO.Fe_2_O_3_.CaO catalysts were prepared using the isovolumetric impregnation method. Iron­(III) nitrate nonahydrate [Fe (NO_3_)_3_·9H_2_O], nickel­(II) nitrate hexahydrate [Ni (NO_3_)_2_·6H_2_O], and calcium oxide (CaO) were added to absolute ethanol at a molar ratio of 1:1:0.8. The mixture was stirred for 1 h and subsequently sonicated for 15 min to ensure uniform distribution of Ni^2+^ and Fe^3+^ species over CaO surface. During mixing, partial hydration of CaO and hydrolysis of metal ions occurred, leading to the formation of surface-bound hydroxide species. After mixing, the solution was kept for 24 h, then dried at 105 °C for 12 h in the oven to remove the ethanol, yielding a solid precursor, followed by calcination at 500 °C in a muffle furnace. During calcination, the metal nitrates decomposed to form NiO and Fe_2_O_3_, while Ca (OH)_2_ reverted to CaO.^13–16^ The final catalyst, NiO.Fe_2_O_3_/CaO consist of a well dispersed multiphase system comprising of CaO, NiO and Fe_2_O_3_, which was used in the chemical reactions after being ground into a fine powder using a mortar pestle. All reactions were conducted in a batch reactor. The step-by-step preparation of the catalyst is illustrated in Figure 1.

**Figure 1:**
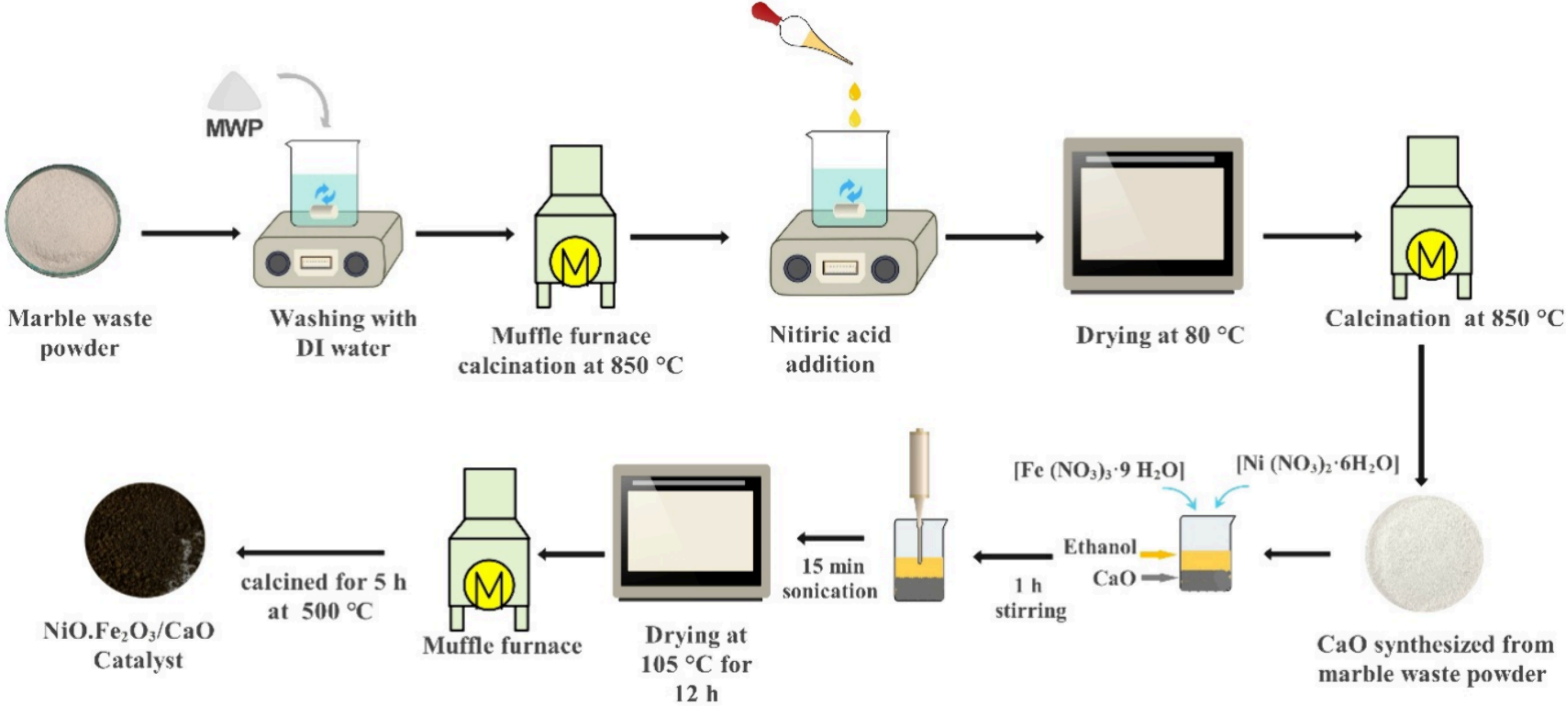
Schematic illustration of the preparation method for the NiO.Fe_2_O_3_/CaO catalyst.

### 2.3 Characterizations of the Catalyst

X-ray diffraction (XRD) analysis was performed using a Panalytical X’Pert Pro diffractometer to determine the crystalline structure of the fresh catalyst. The obtained diffractograms were compared with the JCPDS database for phase identification. Fourier-transform infrared (FTIR) spectra were recorded using a PerkinElmer FTIR spectrometer in the range of 4000 to 400 cm^–1^. Scanning electron microscopy (SEM) coupled with energy-dispersive X-ray spectroscopy (EDS) was employed to examine the catalyst’s morphology and elemental composition. SEM images and EDS mapping were acquired using a Nova Nano SEM 450 (FEI) operated at an accelerating voltage of 15 kV and a working distance of 10 mm. To improve conductivity and reduce charging effects, the samples were sputter-coated with a thin gold layer prior to imaging. The Brunauer–Emmett–Teller (BET) method was used to determine the specific surface area, pore volume, and pore diameter of the catalyst, measured on a Quantachrome NOVA Touch LX2 instrument. X-ray photoelectron spectroscopy (XPS) analysis was carried out on an Omicron ESCA+ system equipped with a monochromatic Al Kα X-ray source and a charge neutralizer. The surface basicity of the catalysts was evaluated by CO_2_ temperature-programmed desorption (CO_2_-TPD) using a Quantachrome chemisorption analyzer. Elemental composition was determined using an X-ray fluorescence (XRF) spectrometer (ElvaX)

### 2.4 Batch Reactor Experimental Setup

Catalytic tests were performed in a 500 mL high-temperature, high-pressure batch reactor fabricated from SS316 stainless steel (Amar Equipment Pvt Ltd., Mumbai, India). A schematic diagram is provided in Figure 2. A predetermined amount of catalyst, ranging from 0.5 to 10 g, along with 80 g of glycerol, was loaded into the reactor. The system was purged with nitrogen for 15 min to establish an inert atmosphere. The reactor contents were then heated to the desired temperature, with reactions performed within the temperature range of 210–270 °C, under the uniform continuous stirring at 450 rpm throughout the experiments. The time required to reach the target temperature typically ranged from 15 to 30 min. Following the reaction, the reactor was cooled and depressurized, and samples were collected for further analysis. Reaction times were varied from 15 to 210 min to study the reaction mechanism and monitor the formation and progression of various products. Glycerol conversion in the presence of the catalyst resulted in both liquid products, mainly organic acids and propanediol, and gaseous products, indicating partial transformation of glycerol into gas-phase species.

**Figure 2:**
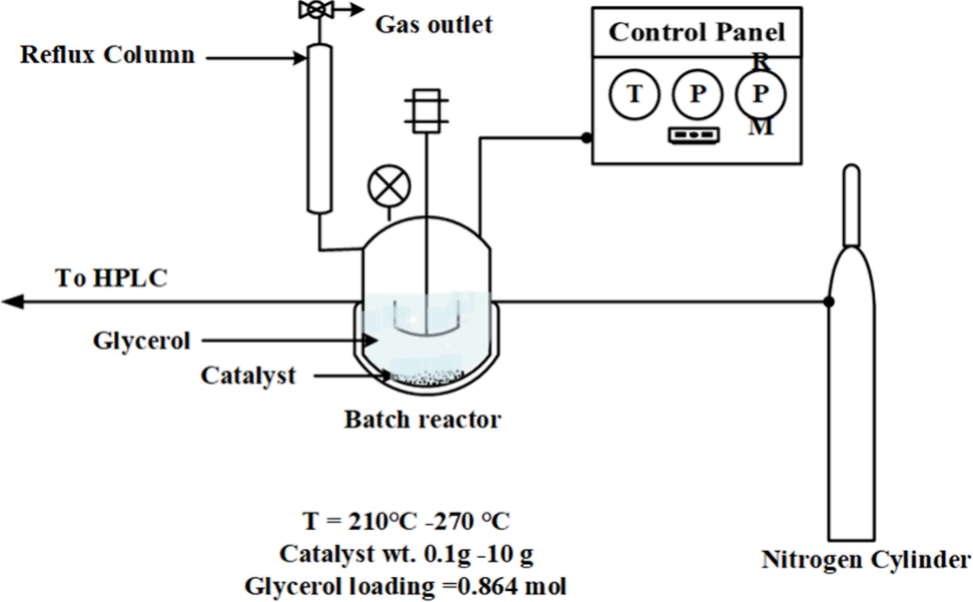
Schematic diagram of batch reactor SS316.

### 2.5 Product Analysis

After completion of the reaction, the catalyst was separated from the product mixture by centrifugation using a centrifuge (REMI RM 12CL) at 12,000 rpm for 15 min. The resulting liquid phase was then filtered through a 0.45 μm syringe filter and diluted 10-fold prior to analysis by high-performance liquid chromatography (HPLC). An injection volume of 25 μL was used in the HPLC system (Shimadzu Nexera XR, Japan), with 0.5 mM H_2_SO_4_ as the mobile phase. Organic acids were detected using a photodiode array (PDA) detector, while glycerol and other products were analyzed with a refractive index detector (RID). The flow rate was maintained at 0.6 mL/min at ambient temperature. Separation was performed on a Bio-Rad Aminex HPX-87H column (300 mm × 7.8 mm) with 9 μm particle size, operated at 30 °C. Representative chromatograms are shown in Figure 3. Glycerol conversion and organic acid yields were calculated according to eqs 5 and 6.

$$\%conversion=\frac{\left[Initialmolesofglycerol-finalmolesofglycerol\right]}{Initialmolesofglycerol}\times 100$$ 5

$$\%Productyield=\frac{Molesofproductformed}{Initialmolesofglycerol}\times 100$$ 6

**Figure 3:**
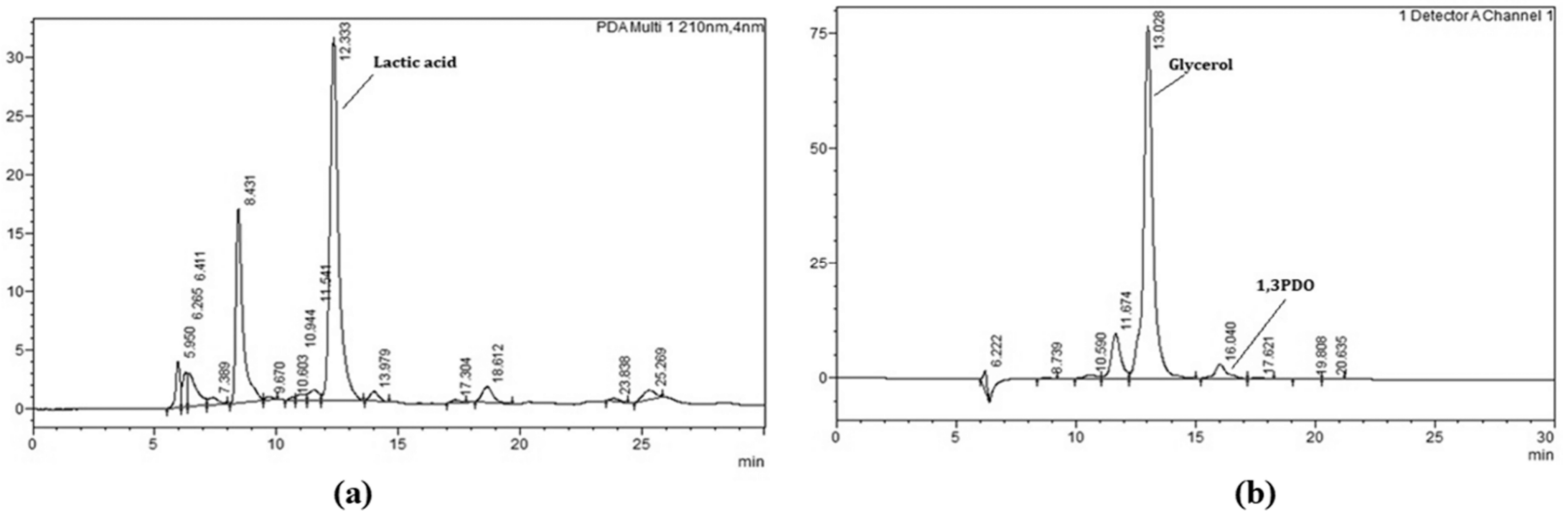
HPLC chromatogram of reaction mixture using (a) PDA and (b) RID detector.

## 3 Results and Discussion

### 3.1 Powder XRD Analysis

PXRD was used to examine the synthesized catalyst’s degree of crystallization, including its composition, crystal structure, and particle size. The XRD pattern of the catalyst is shown in Figure 4. The sharp diffraction peaks indicate a high degree of crystallinity in the catalyst. For NiO.Fe_2_O_3_/CaO catalyst, the most intense peaks appeared at 2θ values of 18.4° (0 0 1), 29.9° (1 3 1), 32.4° (1 1 1), 33.5° (2 0 0), 34.6° (0 2 2), 37.7° (0 2 0), 43.7° (0 2 0), 54.2° (0 2 2), and 63.3° (1 1 2). The observed phases were matched with JCPDS card numbers for CaO (96–900–8606), Ca (OH)_2_ (96–100–1789), NiO (96–152–6381), and Ca_2_Fe_2_O_5_ (96–722–9417).

**Figure 4:**
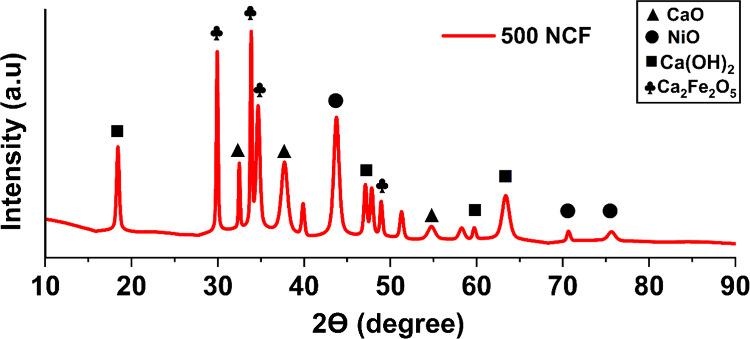
XRD analysis of NiO.Fe_2_O_3_/CaO catalyst.

Debye- Scherer eq 7 was used to find out the average crystalline size of the catalyst NiO.Fe_2_O_3_/CaO.

$$D=\frac{k\lambda }{\beta \cos\theta }$$ 7

Where D is the average crystallite size, K is the dimensionless shape factor, λ is the X-ray wavelength, θ is the Bragg’s angle, and β is the full width at half-maximum (fwhm) of the diffraction peak. From eq 7, the average crystalline size of NiO.Fe_2_O_3_/CaO was found to be to be 24.7 nm. Crystallite size reflects the available surface area for chemical reactions; smaller crystallite sizes provide a larger surface area, enhancing the catalyst’s reactivity.^17^

### 3.2 FTIR Analysis

The analysis of the functional group present in the catalyst NiO.Fe_2_O_3_/CaO was confirmed by FT-IR. The IR spectrum of NiO.Fe_2_O_3_/CaO is shown in Figure 5. The spectrum was recorded at 400 cm^–1^ to 4000 cm^–1^ as shown in Figure 5. The most intense adsorption bands for the catalyst NiO.Fe_2_O_3_/CaO were found at 3442.64 cm^–1^, 2426 cm^–1^, 1383.81 cm^–1^, 1134.18 cm^–1^, 824.41 cm^–1^ and 577.45 cm^–1^. The broad bands at 3442.64 cm^–1^ and 3639.17 cm^–1^ correspond to O–H stretching vibrations, attributed to adsorbed water molecules on the catalyst surfaces.^18^ The peak at 2426.09 cm^–1^ in the NiO.Fe_2_O_3_/CaO catalyst is assigned to O–C–O functional groups. The peak at 1383.81 cm^–1^ corresponds to the symmetric stretching of carbonate species (CO_3_^2–^), likely originating from CaCO_3_ or adsorbed CO_2_ on the catalyst surface. The low wavenumber bands at 824.40 and 577.45 cm^–1^ are characteristic of metal–oxygen vibrations, confirming the incorporation of Ni and Fe into the CaO lattice and the formation of mixed metal oxides.

**Figure 5:**
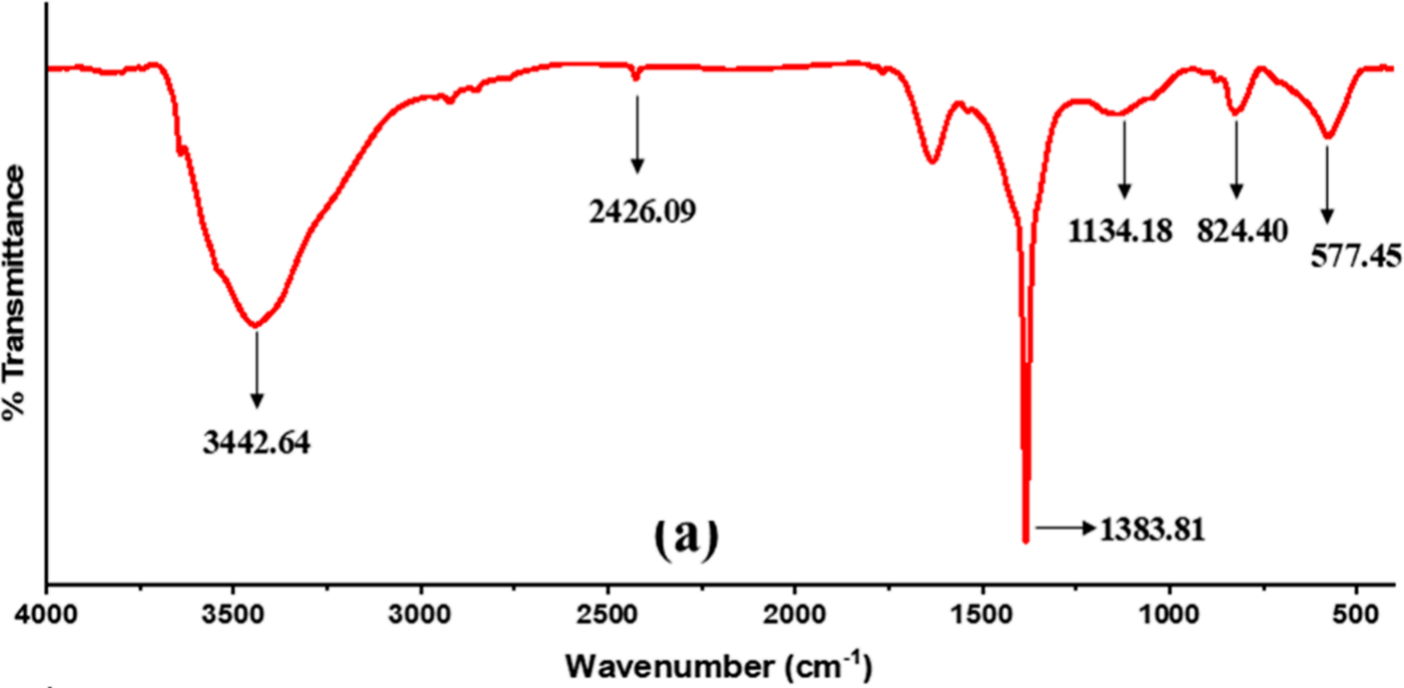
FTIR of NiO.Fe_2_O_3_/CaO catalyst.

### 3.3 SEM-EDS

The topology of the calcined NiO.Fe_2_O_3_/CaO catalyst is presented in Figure 6. SEM imaging was performed at magnifications ranging from 2 to 200 μm to capture both the microstructural features and overall morphology of the catalyst surface. As shown in Figure 6(a), thin, elongated, thread-like shapes are clearly visible at 2 μm magnification. Similarly, Figure 6(b) shows a uniform distribution of these elements throughout the sample, indicating good homogeneity. The EDS spectrum gives the elemental composition of the catalysts as shown in Figure 6(c). The EDS results confirm the presence of Ni, Ca, Fe, and O in their oxide forms, consistent with the XRD findings. Quantitative EDS analysis indicated an average composition of 53.48 wt % Ca, 26.47 wt % Ni, 13.82 wt % Fe, and 6.23 wt % O.

**Figure 6:**
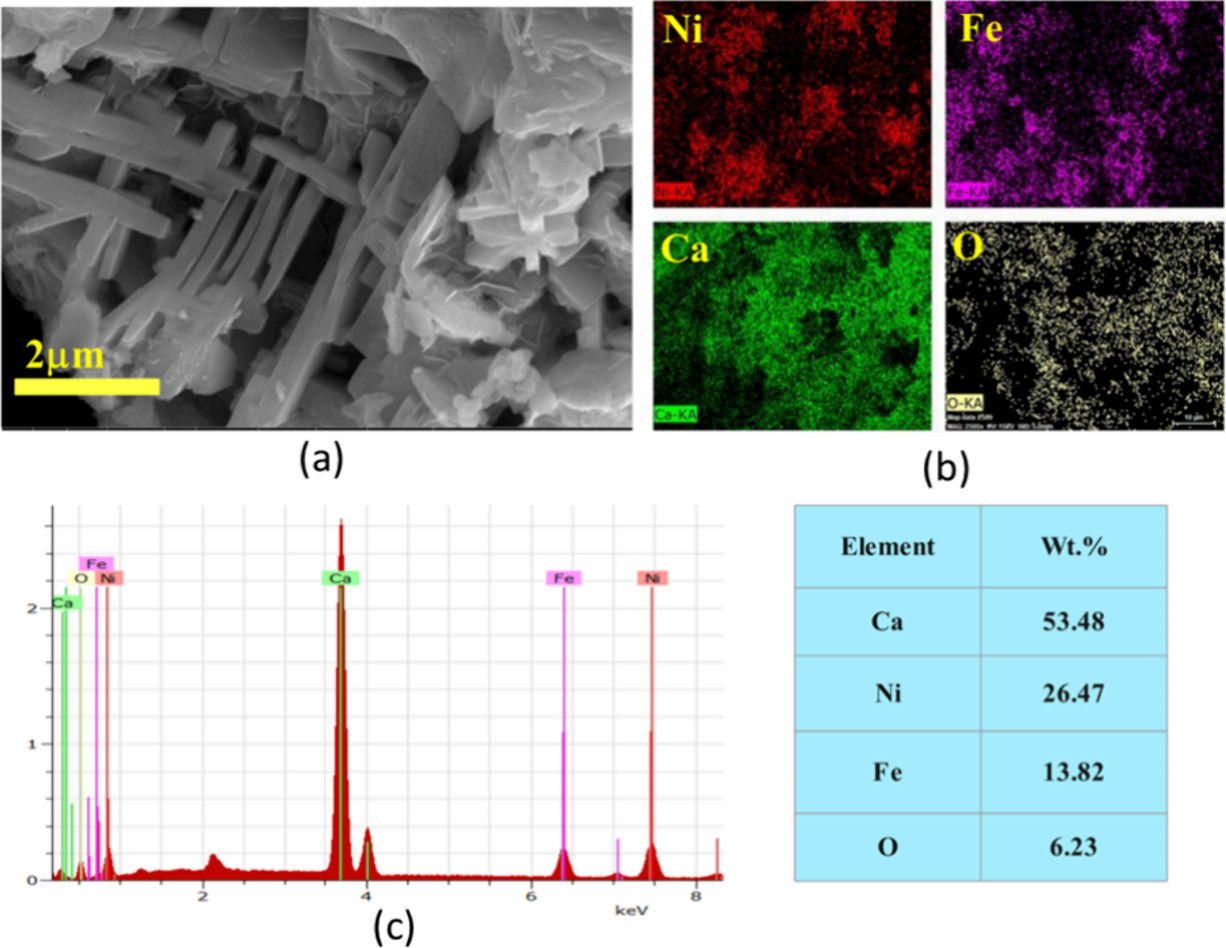
SEM images of the NiO.Fe_2_O_3_/CaO catalyst showing (a) filament-type morphology; (b) homogeneous elemental distribution; (c) EDS mapping showing elemental composition.

### 3.4 TEM Analysis

TEM analysis was performed to study the obtained catalyst’s structural and morphological features, as shown in Figure 7. In the inset, the particle size distribution is shown, obtained by measuring 100 individual particles from the TEM image, with an average particle diameter of 10.56 nm. As illustrated in Figure 7(b) (magnified at 50 nm), some catalyst particles exhibit cuboid and irregular morphologies. The high-resolution TEM image also reveals lattice fringes of the NiO.Fe_2_O_3_/CaO catalyst, with an interplanar spacing measured at 0.112 nm as shown in Figure 7(c).

**Figure 7:**
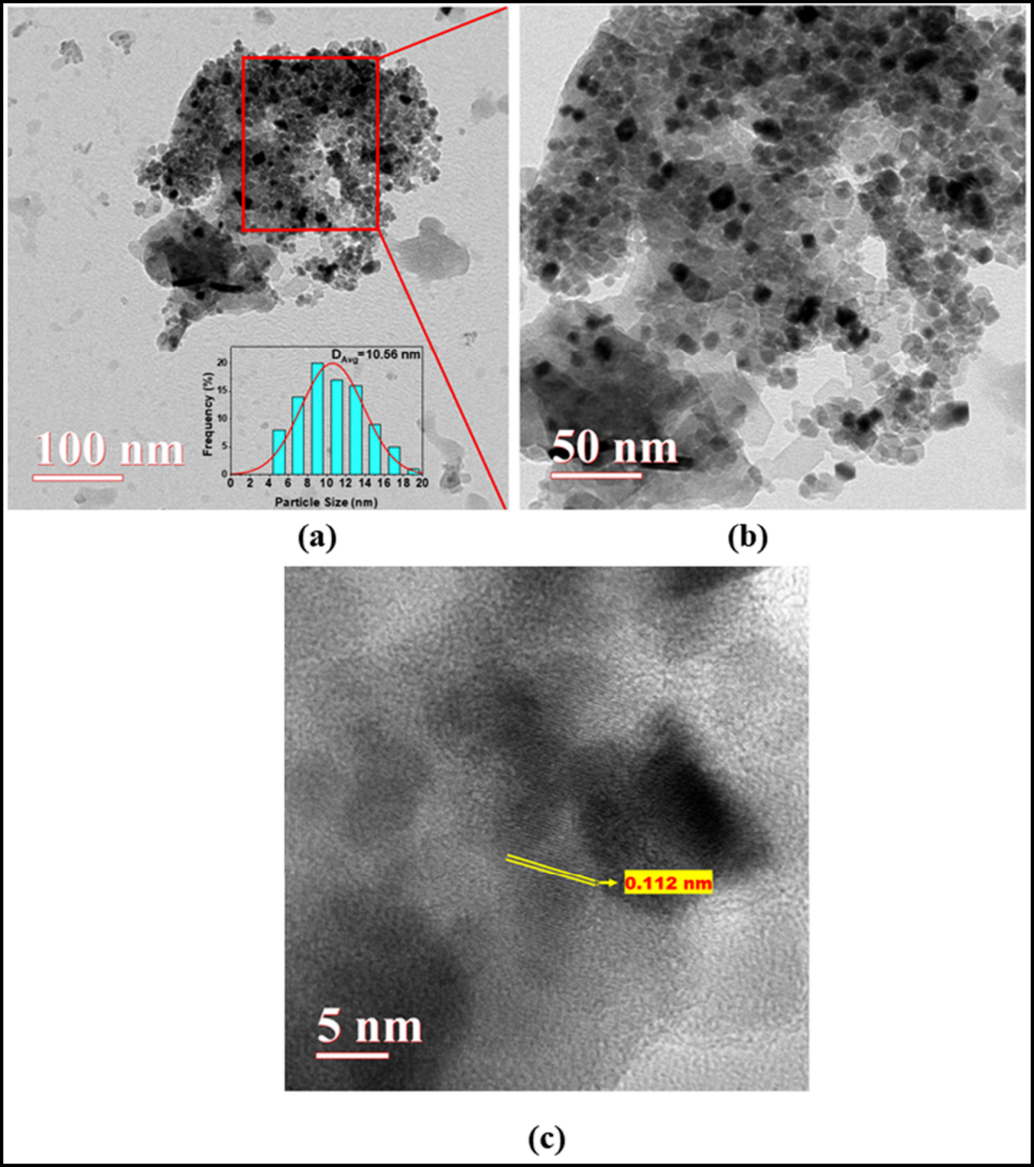
TEM images of the NiO.Fe_2_O_3_/CaO catalyst at different magnifications: (a) 100 nm scale showing overall morphology and particle size distribution, (b) 50 nm scale providing a closer view of the catalyst particles, and (c) high-resolution image displaying the lattice fringes.

### 3.5 BET

For the BET analysis, the samples were degassed under vacuum at 300 °C for 3 h. Nitrogen adsorption–desorption isotherms were then recorded at – 196 °C. The BET method was used to calculate the average pore diameter, specific surface area, and total pore volume, while the Barrett–Joyner–Halenda (BJH) method was applied to evaluate the pore size distribution and total pore volume. The nitrogen adsorption–desorption isotherm of the NiO.Fe_2_O_3_/CaO catalyst is shown in Figure 8. The isotherm exhibits a typical type II curve with a distinct hysteresis loop H3, characteristic of mesoporous materials, consistent with literature reports.^19^ BET analysis revealed a specific surface area of 3.80 m^2^g^–1^, a total pore volume of 0.00471 cm^3^g^–1^, and an average pore diameter of 3.39 nm as shown in Figure 8(a). Figure 8(b) shows that the material displays mesoporosity with a dominant pore radius around 2.8 nm. The differential pore volume (dV­(r)) decreases with increasing pore size, indicating a relatively narrow pore size distribution, an attribute favorable for catalytic applications. The total pore volume was confirmed by integrating dV­(r) over the full pore radius range, further supporting the mesoporous nature of the catalyst.

**Figure 8:**
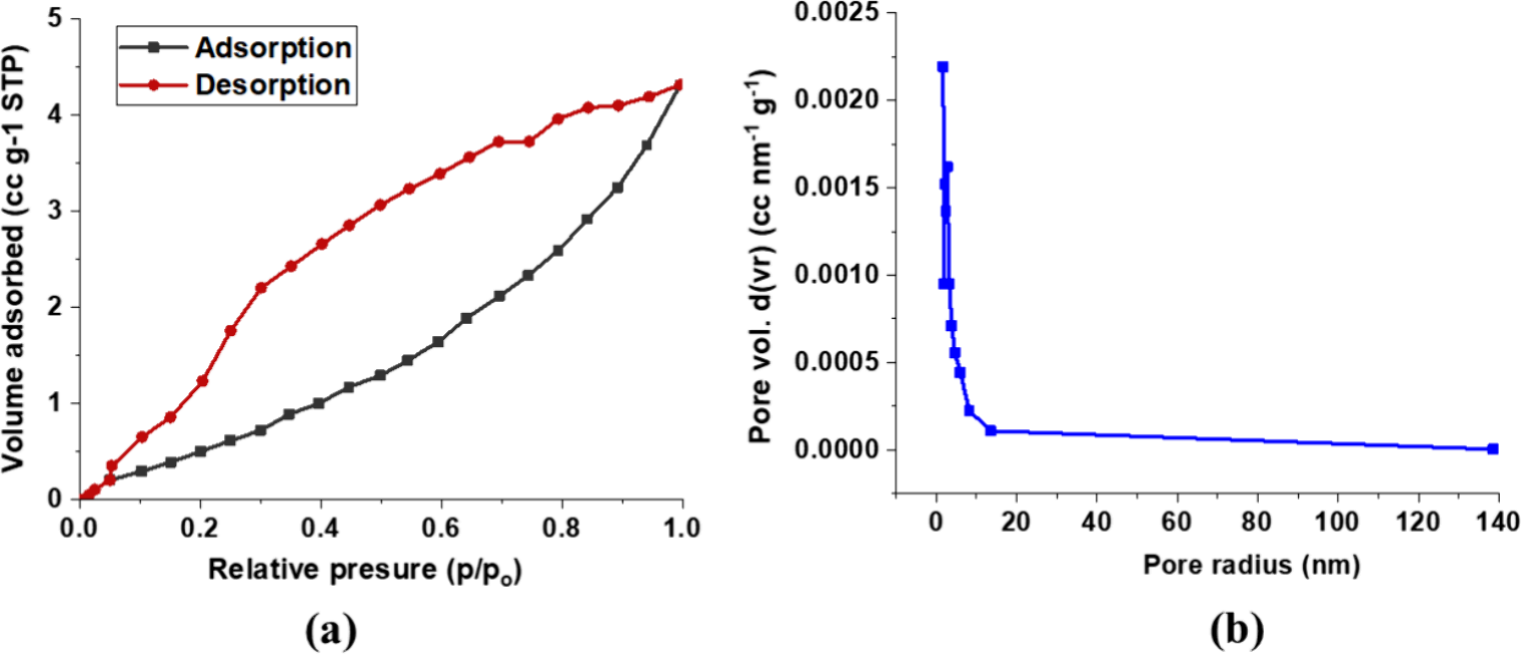
(a) The nitrogen adsorption–desorption isotherm (b) pore size distribution of NiO.Fe_2_O_3_/CaO.

### 3.6 XPS Analysis

The XPS analysis of the catalyst NiO.Fe_2_O_3_/CaO presented in the Figure 9(a–d) confirms the successful incorporation and oxidation states of Fe, Ni, Ca and O in the synthesized catalyst. In Figure 9(a), the Fe 2p spectrum exhibits two major peaks at binding energies of approximately 711.2 and 724.3 eV, attributed to Fe 2p^3/2^ and Fe 2p^1/2^, respectively, indicating the presence of Fe^3+^ species.^20^ Figure 9(b) shows the Ni 2p spectrum, where peaks observed at 856.64 and 873.56 eV correspond to Ni 2p^3/2^ and Ni 2p^1/2^, respectively. A weak satellite feature around 875 eV further supports the presence of Ni^2+^ in the catalyst matrix.^21,22^ In Figure 9(c), the Ca 2p spectrum displays two distinct peaks at 347.6 eV (Ca 2p^3/2^) and 351.1 eV (Ca 2p^1/2^), consistent with the presence of Ca^2+^ in the form of CaO. Figure 9(d) presents the O 1s spectrum, which was deconvoluted into two distinct components. The dominant peak at approximately 532.5 eV (O 1s_1_) is attributed to surface adsorbed oxygen species or hydroxyl groups (O/OH), while the peak at 529.8 eV (O 1s_2_) corresponds to lattice oxygen (M–O) associated with the metal oxide phases present in the catalyst. The relatively higher contribution of the adsorbed oxygen component suggests a hydroxyl-rich surface with abundant oxygen vacancies or defect sites, which are known to play a critical role in enhancing catalytic activity. Collectively, these results confirm the effective incorporation of Fe^3+^, Ni^2+^, Ca^2+^, and their associated oxygen species within the catalyst framework, these results confirm the effective incorporation of the active metal species within the catalyst, essential for its expected performance in glycerol conversion.

**Figure 9:**
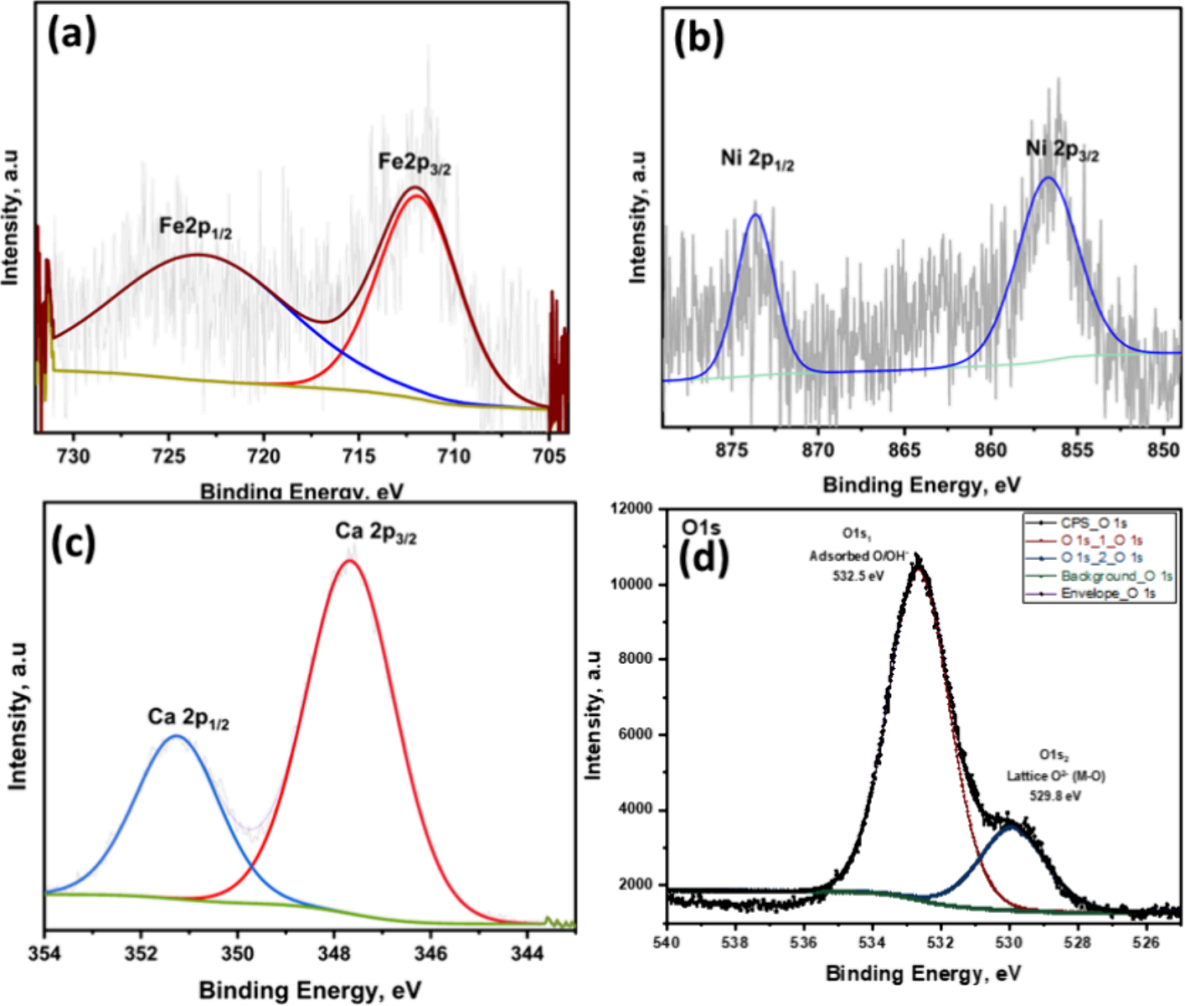
XPS spectra of the catalyst NiO.Fe_2_O_3_/CaO showing (a) Fe 2p, (b) Ni 2p, (c) Ca 2p and (d) O1S regions, confirming the incorporation of Fe^3^^+^, Ni^2^^+^, and Ca^2^^+^ as their respective metal oxides and revealing the coexistence of surface adsorbed and lattice oxygen species.

### 3.7 CO_2_-TPD

The surface basicity of the catalyst NiO.Fe_2_O_3_/CaO was evaluated by CO_2_ temperature-programmed desorption (CO_2_-TPD) using a Quantachrome ChemBET Chemisorption Analyzer. Approximately 0.020 g of catalyst was loaded into a quartz U-tube reactor. Prior to CO_2_ adsorption, the sample was degassed under a flow of pure helium (80 mL min^–1^) at 150 °C for 1 h to remove physically adsorbed species and moisture. After pretreatment, the sample was cooled to 50 °C, and CO_2_ was introduced for 30 min to allow adsorption on the basic sites of the catalyst. Subsequently, the system was purged with helium to remove weakly physiosorbed CO_2_. The CO_2_-TPD analysis was then carried out by heating the sample from 50 to 800 °C at a heating rate of 20 °C min^–1^ under a helium flow (80 mL min^–1^), and the desorbed CO_2_ was continuously monitored using a thermal conductivity detector (TCD). The amount of desorbed CO_2_ was quantified using a calibration injection file, enabling the determination of the surface basicity of the catalysts.

The surface basicity of the NiO.Fe_2_O_3_/CaO catalyst was evaluated by CO_2_ temperature-programmed desorption, and the corresponding desorption profile is presented in Figure 10. Three high-temperature desorption peaks were observed at 629 °C, 638 °C, and 770 °C, indicating the predominance of strong basic sites. The peaks cantered at 629 and 638 °C are attributed to strong lattice O^2–^ species associated with CaO.^23^ The presence of these high-temperature peaks confirms the highly basic nature of the catalyst surface.

**Figure 10:**
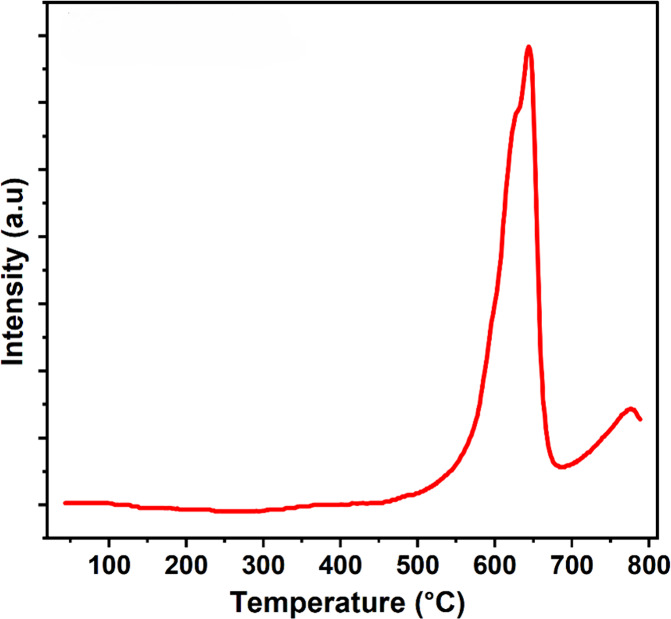
CO_2_ TPD of catalyst NiO.Fe_2_O_3_/CaO.

The desorption peak at 770 °C is assigned to strongly bound carbonate species formed during CO_2_ adsorption.^24^ The total basicity of the NiO.Fe_2_O_3_/CaO catalyst was calculated to be 1.505 mmol g^–1^, demonstrating a high density of strong basic sites. Such strong O^2–^ lattice sites are favorable for glycerol activation via deprotonation of hydroxyl groups, thereby facilitating the formation of glyceroxide intermediates and promoting the catalytic conversion of glycerol.^25^

### 3.8 XRF Analysis

The chemical composition of the synthesized catalyst NiO.Fe_2_O_3_/CaO was determined by X-ray fluorescence (XRF) analysis. As shown in Table 1, the catalyst is primarily composed of CaO (61.85 wt %), indicating that the material is predominantly CaO-based and consistent with the use of calcium-rich precursors. Nickel is present at 16.47 wt %, which serves as the active metallic phase responsible for catalytic activity. Fe_2_O_3_ is also present in a significant amount (14.13 wt %), suggesting its role as a promoter that can influence metal dispersion and catalytic stability. The high CaO content further implies the presence of strong basic sites, which are beneficial for glycerol conversion reactions. Other elements were present only in trace amounts (<1 wt %) and thus, there effect on the reaction was omitted and not considered in this work.

**Table 1:** XRF Analysis of the Catalyst NiO.Fe_2_O_3_/CaO

Compounds	Concentration
CaO	61.85%
Fe_2_O_3_	14.13%
Ni	16.47%

### 3.9 Effect of Catalyst Loading on Lactic Acid Yield and Byproduct Formation

The effect of catalyst loading on product distribution was investigated using the NiO.Fe_2_O_3_/CaO catalyst, with loading varied from 0.5 to 10 g and the results are shown in Figure 11. On increasing the catalyst weight from 0.5 to 10 g, the conversion of glycerol was significantly increased from 10.34% to 81%. Over the same range of catalyst amount, the corresponding yield of lactic acid was found to be increased from 0.91 mol % to 18.1 mol %, the yield of acetic acid increased from 0.2 mol % to 1.8 mol %, and the yield of formic acid increased from 0.79 mol % to 2.2 mol %. These trends indicated that higher catalyst loading not only enhanced the glycerol conversion but also promotes the formation of lactic acid and low-molecular-weight organic acids.

**Figure 11:**
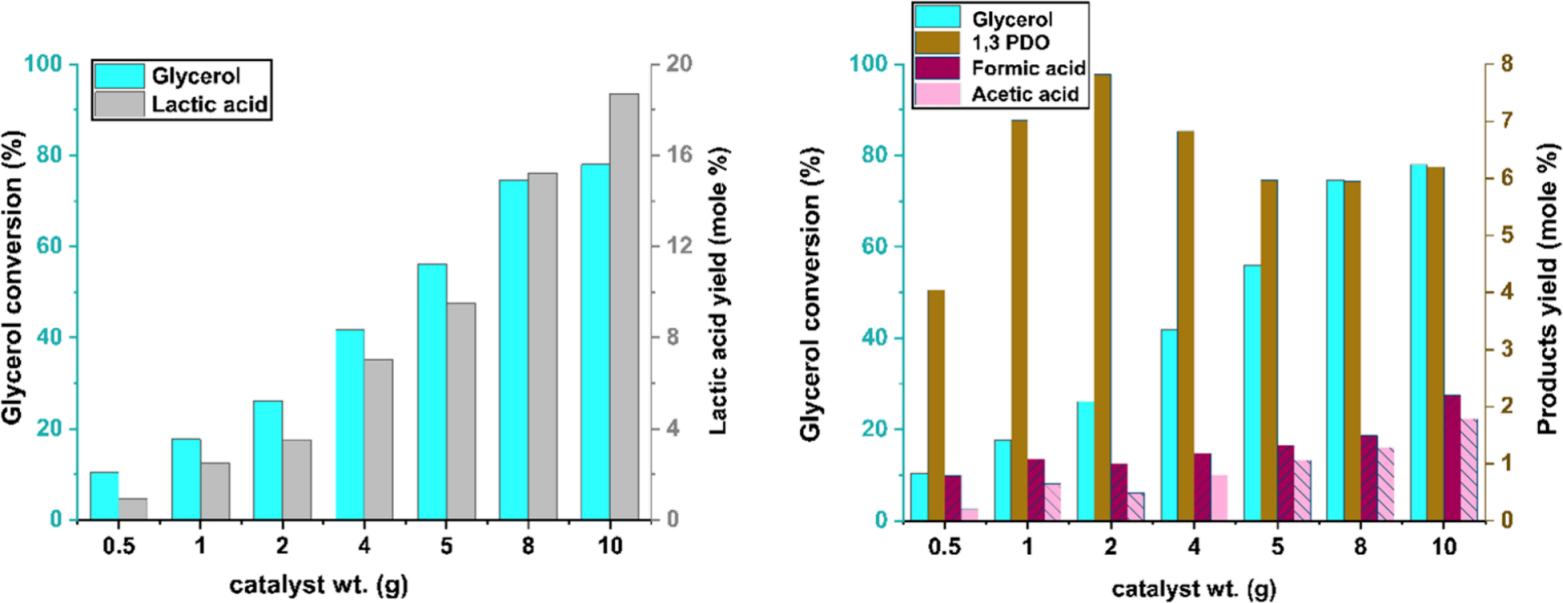
Effect of NiO.Fe_2_O_3_/CaO catalyst loading on glycerol conversion and product yields. Reaction conditions: temperature = 250 °C, reaction time = 180 min, stirring speed = 450 rpm, initial glycerol = 80 g (≈ 0.864 mol).

In the case of 1,3-PDO, the yield increased from 4.04 mol % at 0.5 g to a maximum of 7.82 mol % at 2 g of catalyst. However, further increases in catalyst weight beyond 2 g did not significantly improve 1,3-PDO yield, which remained relatively constant around 6 mol % between 5 and 10 g. While increasing catalyst loading enhances glycerol conversion and yields of various organic acid, the 1,3-PDO yield reaches a plateau at 2 g of catalyst. This may indicate that either the formation of intermediates leading to 1,3-PDO becomes limiting, or that the specific active sites responsible for 1,3-PDO production are already saturated. Moreover, at higher catalyst concentrations, competitive reaction pathways, such as those favoring lactic acid formation or the generation of gaseous byproducts, may become more dominant, thereby constraining the further formation of 1,3-PDO.

Similar observations have been reported in literature. For instance, on increasing the amount of Pd/C catalyst from 0.2 to 0.8 g, the glycerol conversion was found to increase from 93.9% to 99.4%, with minimal change in lactic acid selectivity.^26^ In another study, a Co/CeO_2_ catalyst exhibited a low glycerol conversion (<8%) at 2 wt % loading, but at 10 wt % loading, both glycerol conversion and lactic acid yield improved significantly, with the yield reaching 45 mol % under batch conditions at 160 °C, 2.0 MPa N_2_ pressure, and 4.5 h of reaction time.^27^

These findings highlight that catalyst dosage is a critical parameter influencing glycerol conversion and product selectivity. Specifically, increasing catalyst loading favors the formation of lactic acid and short-chain carboxylic acids (formic and acetic acids), offering a potential strategy for directing product distribution in glycerol valorization pathways.

### 3.10 Effect of Temperature on Lactic Acid Yield and Byproduct Formation

The effect of reaction temperature on glycerol conversion and product distribution was studied by varying the temperature from 210 to 270 °C. As the temperature increased, glycerol conversion improved significantly from 15.37% to 86%. The yield of lactic acid was also increased with temperature, and reached to a maximum of 19.94 mol % at 260 °C, up from 6.91 mol % at 210 °C. However, a further increase in temperature to 270 °C resulted in a sharp decline in lactic acid yield to 7.19 mol %, as shown in Figure 12. A similar trend was also observed for 1,3-PDO, in which the yield increased from 1.88 mol % at 210 °C to 8.45 mol % at 260 °C, before decreasing at higher temperatures. The yields of formic acid and acetic acid followed a similar trend, with steady increase with temperature and reached to their maximum yield at 260 °C. Specifically, the yield of formic acid increased from 1.59 to 2.2 mol %, while the yield of acetic acid rose from 0.93 to 1.79 mol % as the temperature increased from 210 to 260 °C. However, both yields decreased when the temperature was further raised to 270 °C. These results indicate that 260 °C was found to be an optimum temperature based on the maximum product yield with higher glycerol conversion. This reduction in lactic acid and 1,3-PDO yields beyond 260 °C is attributed primarily to thermal degradation and secondary decomposition of these products under elevated thermal conditions. At temperatures exceeding 260 °C, these organic acids and diols undergo cracking, dehydration, and further oxidation reactions, which lead to the formation of smaller molecules and byproducts. Specifically, they can convert into light gases such as CO, CO_2_, H_2_, and CH_4_, as well as smaller oxygenated compounds like formaldehyde and acetaldehyde.^28–31^ Additionally, dehydration reactions produce water, and under severe conditions, carbonaceous deposits (coke) may form on the catalyst surface. These secondary reactions reduce the selectivity toward lactic acid and 1,3-PDO at elevated temperatures, beyond 260 °C. These observations align with previous studies where temperature was identified as a key factor influencing product distribution in glycerol valorization. Shanqi Li et al.^30^ conducted the selective oxidation of glycerol to lactic acid using a CuO/activated carbon catalyst. They observed that the lactic acid yield increased with rising temperature from 150 °C to a maximum of 73.85% at 190 °C. However, further increasing the temperature to 200 and 210 °C led to a decline in yield, which was attributed to the occurrence of side reactions and further oxidation of lactic acid.

**Figure 12:**
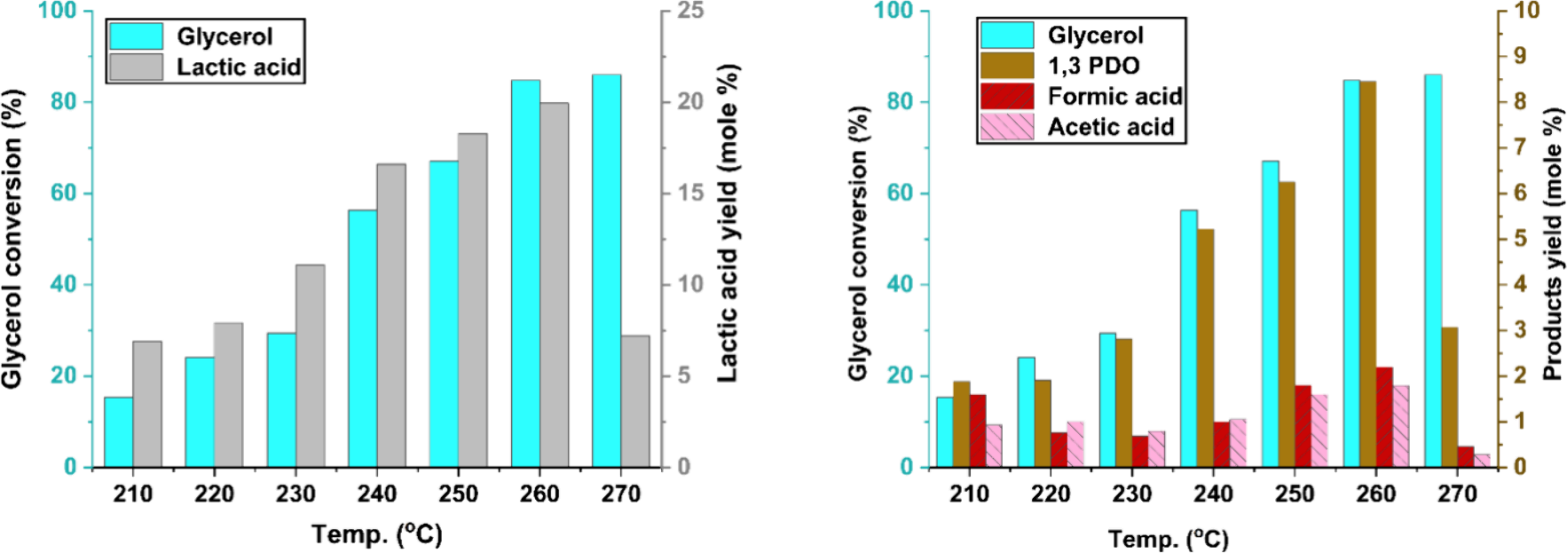
Effect of temperature on product formation using NiO.Fe_2_O_3_/CaO catalyst. Reaction conditions: catalyst weight = 10 g, reaction time = 180 min, glycerol loading = 80 g (0.864 mol), stirring speed = 450 rpm.

### 3.11 Effect of Time on Lactic Acid Yield and Byproduct Formation

The effect of reaction time on glycerol conversion and the distribution of major products was systematically studied at a fixed temperature of 260 °C and a catalyst loading of 10 g. The results are shown in Figure 13. Glycerol conversion showed a consistent upward trend with increasing reaction time, rising from 31.12% at 15 min to a maximum of 91.24% at 210 min, indicating enhanced catalytic activity and progressive substrate depletion over time. The yield of lactic acid, the primary product, increased from 14.5 mol % at 15 min to a maximum of 20.72 mol % at 150 min, suggesting that moderate reaction durations favor lactic acid formation. Beyond this point, a slight decline to 17.43 mol % at 210 min was observed, which may be attributed to thermal degradation, competitive side reactions, or further transformation of lactic acid under extended thermal exposure. In a related study on the alkaline hydrothermal conversion of glycerol to lactic acid conducted in a batch reactor at temperatures ranging from 240 to 300 °C, a similar trend was observed. The lactic acid yield increased with reaction time, reaching a maximum of 85.5 mol % at 90 min. However, with further extension of the reaction time to 240 min, the glycerol conversion remained constant while the lactic acid yield declined to 75 mol %, likely due to secondary degradation processes. This decrease was attributed to the formation of gaseous byproducts such as H_2_, H_2_O, CO_2_, CO, and CH_4_, likely resulting from the further decomposition of lactic acid and other intermediates.^28^

**Figure 13:**
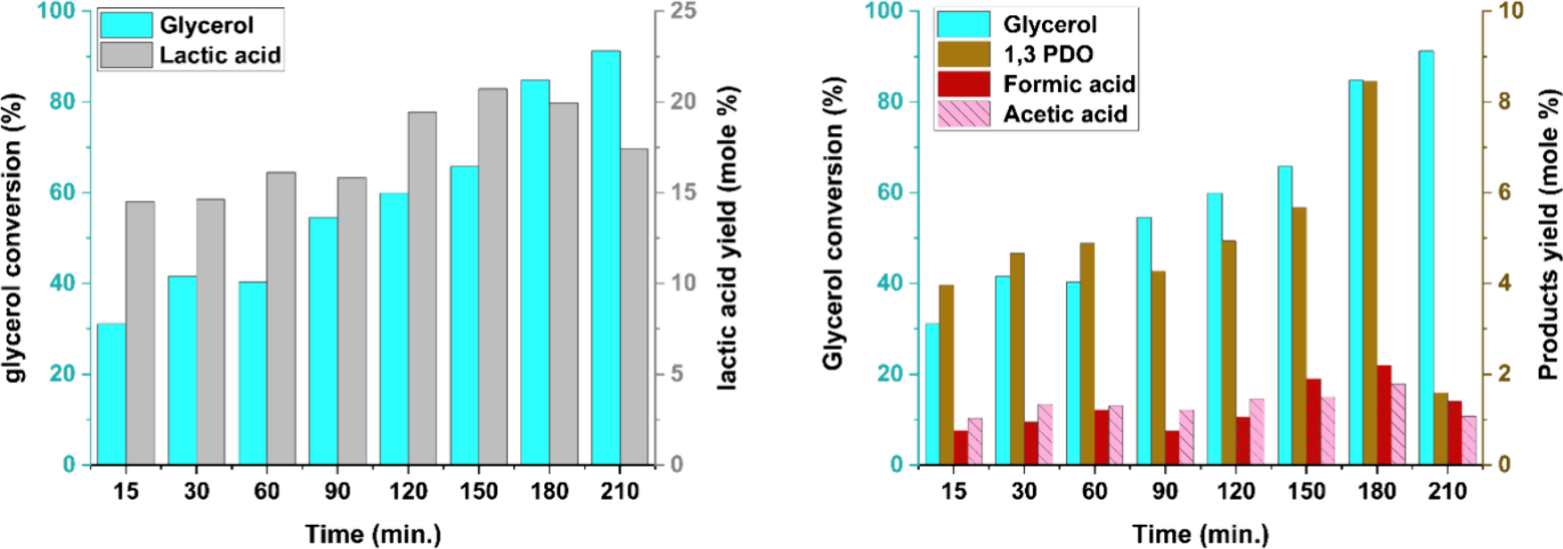
Effect of reaction time on product formation using the NiO.Fe_2_O_3_/CaO catalyst. Reaction conditions: temperature = 260 °C, glycerol loading = 80 g (0.864 mol), catalyst weight = 10 g, stirring speed = 450 rpm.

The production of 1,3-PDO followed a comparable trend, with the yield increasing from 3.96 mol % at 15 min to a maximum of 8.45 mol % at 180 min. A sharp decrease to 1.60 mol % at 210 min indicates that 1,3-PDO is more sensitive to prolonged reaction times, likely due to instability under high-temperature conditions or conversion into secondary products. Similarly, the yields of minor byproducts such as formic acid and acetic acid increased gradually, reaching their highest values of 2.20 mol % and 1.79 mol %, respectively, at 180 min, before slightly declining. These observations collectively suggest that while extended reaction times enhance glycerol conversion, they may not always be favorable for maximizing the yield of specific target products due to thermally induced degradation or over conversion of intermediates. Therefore, an optimal reaction time around 150–180 min is critical for achieving high selectivity toward lactic acid and 1,3-PDO at 260 °C.

## 4 Reaction Mechanism

The conversion of glycerol into value-added chemicals such as lactic acid and 1,3-PDO has garnered significant attention in recent years, with numerous reaction pathways proposed in the literature.^32–36^ These transformations do not proceed via a single, linear route but rather involve a complex network of mechanisms. In particular, dehydrogenation, intramolecular hydride shift, dehydration, and hydrogenation play central roles, as observed in this study. Additional pathways such as isomerization, oxidation, and hydrogenolysis have also been reported in the literature, depending on the catalyst and reaction environment. Each of these steps is strongly influenced by the catalyst composition, reaction conditions, and reactor configuration. The proposed reaction pathway is shown in Figure 14.

**Figure 14:**
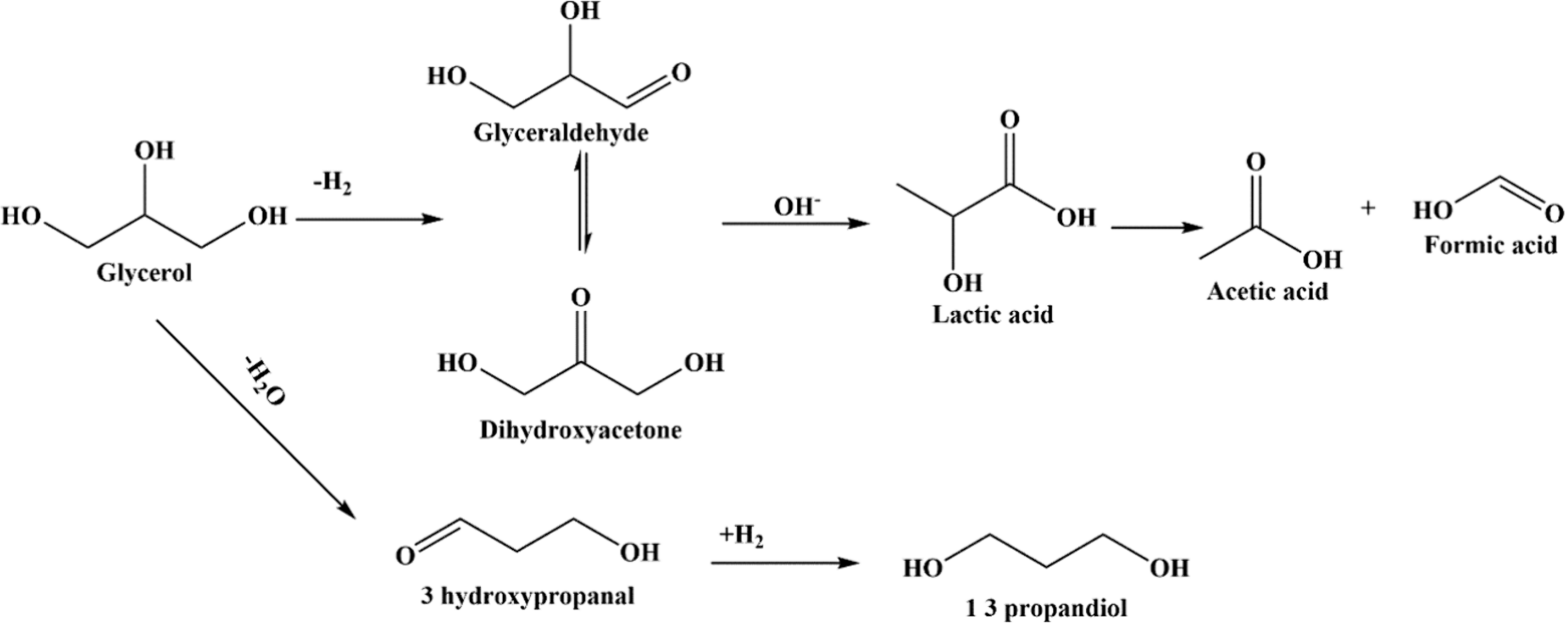
Proposed reaction pathways.

In our study, the multifunctional NiO.Fe_2_O_3_/CaO catalyst played a central role in steering the reaction. When glycerol was introduced to this catalytic system, it first underwent dehydrogenation, giving rise to crucial intermediates like glyceraldehyde and dihydroxyacetone (DHA). These species, reactive and short-lived, marked the divergence into multiple potential pathways. The basic character imparted by calcium oxide facilitated the rearrangement of glyceraldehyde through an intramolecular hydride shift, ultimately forming lactic acid. Meanwhile, the presence of nickel and iron added redox functionality, making it possible to hydrogenate other intermediates and drive the formation of additional products.

Under prolonged reaction times or oxidative environments, lactic acid did not remain stable so it partially degraded into smaller carboxylic acids like acetic acid and formic acid. Along another pathway, glycerol molecules underwent dehydration, yielding 3-hydroxypropanal, which was then converted into 1,3-PDO via metal-assisted hydrogenation, a reaction enabled by the strong hydrogen transfer capabilities of Ni and Fe in the catalyst as shown in Figure 14. These findings underscore the multifunctional nature of the NiO.Fe_2_O_3_/CaO catalyst, which not only drives the main valorization pathways of glycerol but also enables a range of secondary transformations.

The NiO.Fe_2_O_3_/CaO catalyst acts as a multifunctional catalytic system in glycerol valorization due to the synergistic interaction of its metallic and basic components. Ni and Fe provide redox-active sites that facilitate the dehydrogenation of glycerol to glyceraldehyde or dihydroxyacetone (DHA) through C–H and O–H bond activation and hydrogen abstraction via d-orbital interactions. These metals/metal oxides support hydrogenation reactions, in which Ni dissociatively adsorbs H_2_ and, together with Fe in a bimetallic configuration, transfers surface hydrogen species to reduce intermediates, such as 3-hydroxypropanal, to 1,3-propanediol. In contrast, CaO contributes strong basic sites (O^2–^/OH^–^) that promote base-catalyzed transformations, particularly the intramolecular hydride shift of glyceraldehyde to lactic acid through enolate formation and a 1,2-hydride rearrangement. The catalyst also facilitates other parallel reactions: Fe Lewis acid sites promote dehydration of glycerol to 3-hydroxypropanal via −OH activation and water elimination, while the combined action of Ni/Fe redox sites and CaO basicity can induce C–C bond cleavage, leading to the formation of acetic acid and formic acid through oxidative scission and stabilization of carboxylate intermediates. Overall, the cooperative redox, acidic, and basic functionalities of Ni, Fe, and CaO enable multiple reaction pathways involved in glycerol conversion.

## 5 Comparison of the Present Synthesized Catalyst NiO.Fe_2_O_3_/CaO with Other Reported Heterogeneous Catalysts for the Production of Lactic Acid

The activity of the present catalyst in glycerol conversion to lactic acid was compared with several heterogeneous catalysts reported in the literature, as shown in Table 2. The lactic acid yield, glycerol conversion, and reaction conditions such as temperature, reaction time, and catalyst loading were considered as key performance parameters. The activity of the present catalyst in lactic acid production was compared with various heterogeneous catalysts reported in the literature, as summarized in Table 2.

**Table 2:** Lactic Acid Production from Glycerol Using Various Catalysts under Different Operating Conditions^a^

Catalyst	Substrate	Operating parameters	Products Yield (mol %)	Glycerol (conversion %)	ref.
NiO.Fe_2_O_3_/CaO	glycerol	*T* = 260 °C, *t* = 150 min, catalyst wt. = 10g,	Y_LA_= 20.7,	68.5	This study
			Y_1,3 PDO_ = 5.67		
			Y_FA_ = 1.9		
			Y_AA_ = 1.5		
0.25NiO/CaO	glycerol	*T* = 290 °C, *t* = 1.5 h, 15 wt % catalyst loading	Y_LA_ = 41.4	90	^11^
Ag_3_PMo_12_O_40_	glycerol	*T* = 60 °C, *t* = 5 h, cat wt. mmol = 0.023, glycerol solution = 5 mL of 10 wt %	Y_LA_ = 72	89	^39^
Ni–NiOx@C-200	glycerol	*T* = 200 °C, *t* = 30 min, NaOH/glycerol molar ratio = 1.1, catalyst weight = 0.05g	Y_LA_ = 46.8, Y_AA_ = 4.3, Y_FA_ = 9.6, Y_GLA_ = 1.7, Y_MA_ = 17.7	100	^38^
AuPt/TiO_2_	glycerol	*T* = 90 °C, NaOH/glycerol molar ratio = 4, 0.22 mol·L^–1^ glycerol in H_2_O	Y_LA_ = 26	30	^37^
1:1 CeZr/SBA-15	glycerol	*T* = 250 °C, *t* = 2 h, 20 wt % cat loading	Y_LA_ = 22	82.5	^19^

a LA = lactic acid, FA = formic acid, AA = acetic acid, 1,2PDO = 1,2 propanediol,1,3PDO = 13 propanediol, GLA = glyceric acid, MA = methanol S = selectivity, Y = yield.

In this study, the NiO.Fe_2_O_3_/CaO catalyst synthesized from CaO derived from marble waste powder achieved a lactic acid yield of 20.7 mol % with 68.5% glycerol conversion at 260 °C over 150 min using 10 g of catalyst. While this yield is moderate compared to other reported systems, the catalyst simultaneously promoted the formation of 1,3-PDO (5.67 mol %), formic acid (1.9 mol %), and acetic acid (1.5 mol %), indicating a balanced distribution of acidic and redox-active sites. Ahmad Abdullah et al.^11^ reported a higher lactic acid yield of 41.4 mol % with 90% glycerol conversion using a 0.25NiO/CaO catalyst at 290 °C for 1.5 h and a catalyst loading of 15 wt %, although the formation of coproducts was not disclosed. Using noble metal-based systems, Melin Tao et al.^28^ achieved the highest lactic acid yield of 72 mol % with 89% glycerol conversion under significantly milder conditions of 60 °C for 5 h over Ag_3_PMo_12_O_40_, a silver-based polyoxometalate catalyst, although with a longer reaction time. Similarly, AuPt/TiO_2_ produced 26 mol % lactic acid with 30% conversion at 90 °C, likely limited by its lower basicity, while a 1:1 CeZr/SBA-15 catalyst yielded 22 mol % lactic acid with 82.5% conversion at 250 °C over 2 h with a 20 wt % catalyst loading^37^ Although noble metal catalysts such as gold and silver demonstrate superior activity under mild conditions, they are substantially more expensive than the NiO.Fe_2_O_3_/CaO catalyst developed in this work. Furthermore, a Ni–NiO_*x*_@C-200 catalyst reported in the literature achieved complete glycerol conversion with a lactic acid yield of 46.8 mol % at 200 °C in 30 min, coproducing malic acid 17.7 mol %, formic acid 9.6 mol %, acetic acid 4.3 mol %, and glycolic acid 1.7 mol %.^38^ Overall, while the NiO.Fe_2_O_3_/CaO catalyst exhibits moderate selectivity toward lactic acid relative to some high-performance catalysts, its ability to coproduce multiple value-added chemicals such as formic acid, acetic acid, and 1,3-PDO, combined with its cost-effectiveness and sustainability, underscores its suitability as a practical and environmentally benign option for integrated glycerol valorization.

## 6 Conclusion

This study successfully demonstrates the valorization of glycerol into lactic acid using a novel heterogeneous NiO.Fe_2_O_3_/CaO catalyst synthesized from marble waste powder doped with iron and nickel. Under optimized conditions at 260 °C, 150 min, and 10 g of catalyst loading, the catalyst achieved a maximum lactic acid yield of 20.7 mol % with a glycerol conversion of 68.5%. Alongside lactic acid, minor amounts of valuable byproducts such as 1,3-PDO at 5.67 mol %, formic acid at 2.2 mol %, and acetic acid at 1.5 mol % were detected. The multifunctional nature of the NiO.Fe_2_O_3_/CaO catalyst enabled diverse reaction pathways including dehydrogenation, intramolecular hydride shift, dehydration, and hydrogenation, facilitating efficient glycerol transformation.

Compared to other heterogeneous catalysts reported in the literature, the present catalyst operates under relatively moderate reaction conditions while maintaining a favorable balance between catalytic activity and product selectivity, with minimal byproduct formation. While some catalysts yield higher lactic acid production, they often require more severe reaction parameters, longer durations, or expensive materials. In contrast, the marble waste derived NiO.Fe_2_O_3_/CaO catalyst offers a cost-effective, environmentally sustainable, and scalable alternative for glycerol upgrading. These findings underscore its potential as a practical catalyst for sustainable biomass valorisation and contribute to advancing green chemical processes for lactic acid production.
